# Aussie KIDS SAVE LIVES: A position statement from the Australian Resuscitation Council and supported by stakeholders

**DOI:** 10.1111/1742-6723.13840

**Published:** 2021-08-13

**Authors:** Janet Bray, Jason Acworth, Greg Page, Michael Parr, Peter Morley, Australian Resuscitation Council and KIDS SAVE LIVES Stakeholder Organisations

**Affiliations:** ^1^ Australian Resuscitation Council Melbourne Victoria Australia; ^2^ Department of Epidemiology and Preventive Medicine Monash University Melbourne Victoria Australia; ^3^ Prehospital, Resuscitation and Emergency Care Research Unit (PRECRU) Curtin University Perth Western Australia Australia; ^4^ Emergency Department Queensland Children's Hospital Brisbane Queensland Australia; ^5^ Faculty of Medicine The University of Queensland Brisbane Queensland Australia; ^6^ Heart of the Nation Sydney New South Wales Australia; ^7^ Intensive Care Liverpool Hospital Sydney New South Wales Australia; ^8^ Department of Clinical Medicine The University of New South Wales Sydney New South Wales Australia; ^9^ Department of Clinical Medicine Macquarie University Sydney New South Wales Australia; ^10^ Intensive Care The Royal Melbourne Hospital Melbourne Victoria Australia; ^11^ Faculty of Medicine, Dentistry and Health Sciences The University of Melbourne Melbourne Victoria Australia

**Keywords:** basic life support, cardiac arrest, cardiopulmonary resuscitation, children, defibrillation

## Abstract

Every year 25 000 Australians experience a cardiac arrest in our community, but only 12% survive. The faster cardiopulmonary resuscitation and defibrillation, known as basic life support (BLS), is commenced, the greater the chance of survival. Currently, only half of the Australian adults are trained in BLS. The Australian Resuscitation Council and key stakeholder organisations believe that the best way to ensure all Australians know how to save a life is by mandating BLS education and training in our schools. This ‘Aussie KIDS SAVE LIVES’ position statement outlines our strategy to help facilitate the introduction of a programme of regular BLS training into the Australian school curriculum.

## Introduction

**Figure 1:**
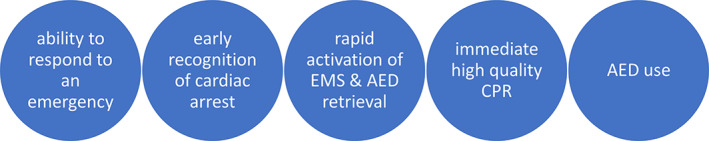
Key concepts of BLS education and training.

*Any Attempt at Resuscitation is Better Than No Attempt* is the maxim of the Australian Resuscitation Council (www.resus.org.au). When deprived of oxygen, brain cells start to die within minutes, hence cardiac arrest is a time‐critical emergency – every second counts. Early cardiopulmonary resuscitation (CPR) and defibrillation, together known as basic life support (BLS) (Fig. 1), provide the greatest chance of survival.^1^

The International Liaison Committee on Resuscitation (ILCOR) was established in 1992 by principal resuscitation organisations to provide a forum to create guidelines to save more lives globally through resuscitation (www.ilcor.org). ILCOR's work initially focussed on the medical science to optimise guidelines for the delivery of high‐quality CPR, but more recently the focus has shifted from *how to best perform CPR* to include recommendations on *how to best teach people to do CPR* and *how to implement systems to facilitate high‐quality CPR*.

Great advances have been made over the last decade in the development of high‐quality CPR delivery and education methods, but when it comes to implementation in our communities, we still have a long way to go.

## OHCA: the current problem in Australia

In Australia, over 25 000 people experience cardiac arrest in the community every year, but only 12% survive.^2^ The majority (80%) of these out‐of‐hospital cardiac arrests (OHCA) occur in our homes, which means that it is usually household members who must recognise that the person is in cardiac arrest and respond to this life‐threatening emergency to maximise survival.

Our emergency response systems are set up so that instructions for CPR and automatic defibrillator (AED) retrieval (if available) can be provided by the dispatcher in emergency calls if cardiac arrest is identified. Despite this, only 40% of Australians in cardiac arrest receive bystander CPR before the ambulance arrives, and only 2% receive community defibrillation.^2^ Examination of emergency calls shows a lack of CPR knowledge is a major barrier.^3^

Anyone can learn how to save a life, yet just over half of Australian adults have received BLS training.^4^ BLS training is associated with an increased willingness and confidence to perform CPR and use an AED.^4^, ^5^ Common barriers to receiving training include a lack of awareness (‘never thought about it’, ‘don't know where to go to learn’), as well as time and cost.^4^, ^5^

### The advantages of BLS education in schools

The Australian Resuscitation Council and key stakeholders believe that the best way to ensure more people are willing and able to respond in an emergency is by ensuring BLS skills are taught to school children annually. BLS training will equip children with lifesaving skills that they will carry through their lives. It also provides a fairer, more equitable and more reliable means of providing training that reaches all Australians. School children and teachers are also important ‘multipliers’ of training, with the value of training extended as they teach their own family and friends.^6^

BLS training has been established as part of the school curriculum in Sweden, France, Denmark, Norway, parts of the United States and, more recently, in the United Kingdom. Early adopters of such programmes have some of the highest bystander CPR and survival rates internationally, indicating that educating children is a successful way to reach the entire population.^7^ For example, in Denmark, the rate of bystander CPR nearly doubled 5 years after CPR training was introduced into schools, with a threefold improvement in survival following OHCA over 10 years.^8^

### The Australian school curriculum

In Australia, the current national curriculum states that children in years 9 and 10 should be able to plan, rehearse and evaluate options for managing situations where their safety, or that of others may be at risk. These options include CPR and first aid. This reference to CPR is the only reference in the national curriculum, implying that children in years prior to 9 and 10 do not necessarily need to be educated about CPR (or BLS skills).

Even though the curriculum for education in Australia is set at a national level, the delivery of the curriculum is decided upon by State and Territory curriculum authorities. There is also scope for further variety in the selection of content and how it is delivered by individual schools, with the ability to tailor the curriculum. The flexible nature of the curriculum allows for each school to cater to the unique needs of the school community and the students, having regard to local contexts, and individual teachers' professional knowledge. Unfortunately, this means that there are no guarantees that a child will be taught CPR or other BLS skills during their schooling in years 9 or 10, let alone at any other stage.

### The KIDS SAVE LIVES initiative

In 2015, a KIDS SAVE LIVES position statement written by key international organisations, including the ILCOR and the European Resuscitation Council, was endorsed by the World Health Organization (WHO). This statement recommends two hours of CPR training annually in schools worldwide.^7^ While this recommendation specifies training should begin in children from 12 years of age, the statement also acknowledges younger children can also be successfully trained.

BLS training can be provided by schools in different formats – using self‐instructive video kits, online and teacher‐led BLS skills in class.^9^ Alternatively, an outside BLS training provider could be engaged to provide training to children.

**Figure 2:**
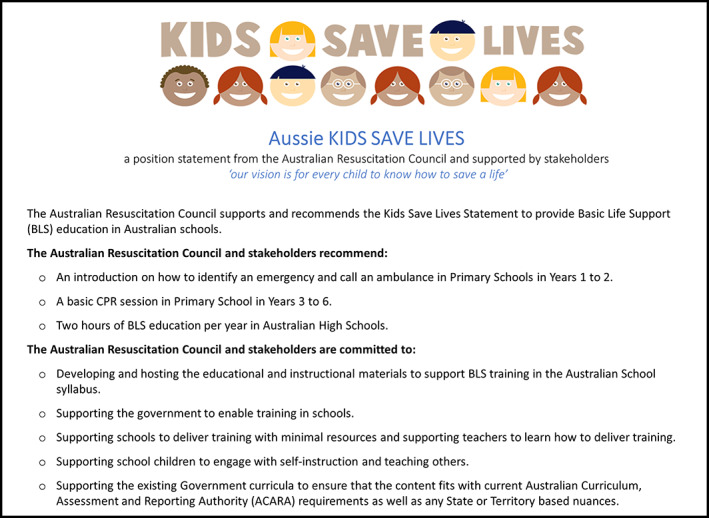
Outline of the Aussie KIDS SAVE LIVES position statement.

Studies have shown that Australian students are supportive of learning BLS training in schools,^10^ and that teachers can be taught to provide training.^7^ The Australian Resuscitation Council and a number of key stakeholder organisations have worked together to formulate a strategy to help facilitate the introduction of a programme of regular BLS training into the Australian school curriculum. This Aussie KIDS SAVE LIVES position statement (Fig. 2) represents the initial step of informing and engaging the community about this important (and lifesaving) initiative.

## Conclusion

The Australian national curriculum is flexible in that it allows for localised and individualised tailoring of its delivery. As a result, there are no guarantees that all Australian school children will leave school having learned BLS, or even CPR without AED use, as a part of their compulsory education. Cardiac arrests can occur anywhere, anytime. Mandating standardised BLS education and training in schools at all levels will increase the number of people equipped with the skills, and concomitantly, the Australian community's ability and preparedness to respond to this life‐threatening emergency. Such a mandate is likely to increase survival rates in cardiac arrest occurring in our community.

## Acknowledgements

The supporting Australian Resuscitation Council members: Anthony Cameron, Carol Carey, Antonio Celenza, Rowena Christiansen, Julie Considine, Ned Douglas, Toni Dunbabin, Kathryn Eastwood, Judith Finn, Elizabeth Flemming‐Judge, Michael Gale, Hugh Grantham, Tracy Kidd, Peter Leman, Helen Liley, Finlay Macneil, Alan Morrison, Michelle Murphy, Kevin Nation, Margaret Nicholson, John Pearn, Craig Ray, Michael Reade, Christopher Scarff, Tony Scott, Tony Smith, Dion Stub, Lakshmi Sunderasan, Marta Thio. Kevin Nation. The supporting stakeholder organisations: Heart of the Nation, Australian and New Zealand College of Anaesthetists, Australian Red Cross, Advanced Paediatric Life Support Australia, Australasian College for Emergency Medicine, Australasian College of Paramedicine, College of Emergency Nursing Australasia, Council of Ambulance Authorities, Royal Australasian College of Surgeons, Surf Life Saving Australia, St John Ambulance Australia, Ambulance Tasmania, Ambulance Victoria. Other supporting individuals: Andrew Lockey (UK).

### Competing interests

None declared.
